# A qualitative study exploring critical care survivors’ and their clinicians’ shared experiences of navigating a fragmented care system

**DOI:** 10.1038/s41598-026-42385-3

**Published:** 2026-03-04

**Authors:** Saira Nazeer, Georgia Mathieson, Zudin Puthucheary, Timothy J. Stephens

**Affiliations:** 1 Adult Critical Care Unit, Royal London Hospital, London, UK; 2 William Harvey Research Institute, Barts and The London School of Medicine and Dentistry, London, UK

**Keywords:** Critical care recovery, Clinical decision making, Integrated health and social care, Health care, Medical research

## Abstract

Recovery following a critical illness is challenging. Follow-up clinic provision is standard of care, but the effectiveness of services remain unclear, with some data demonstrating limited or potential detrimental effects. While the patients’ perspective has been described previously, no studies combine with clinician experience. We explored this combined experience to understand modifiable factors in effective delivery of critical care follow-up services. We conducted a single-centre qualitative interview study in a large UK adult critical care service as part of an evaluation of a pilot follow-up service. Semi-structured interviews with sixteen patients and seven clinicians were analysed using reflexive thematic analysis. Patients experienced uncertainty, fragmented care, and psychological distress as immediate, ongoing challenges marked by a lack of guidance and support. Clinicians described the same broad issues but perceived them somewhat differently, as reflecting the inherent unpredictability of recovery, and caused by delayed presentation of needs, and the constraints imposed by complex care systems. By combining patient and clinician perspectives, our study identifies mismatches in expectations, timing of support, and perceived responsibility. Our data highlight the need for systemic improvements in follow-up care and the importance of integrated social and healthcare services to ensure that patients receive timely and comprehensive support.

**Supplementary Information:**

The online version contains supplementary material available at 10.1038/s41598-026-42385-3.

## Introduction

Over 200,000 people are admitted to critical care every year in the United Kingdom (UK)^1^. Mortality for patients who suffer a critical illness continues to decrease, as a result of advances in medicine and healthcare, with 70% of patients surviving to leave hospital^2,3^. However, for many, this is the start of the recovery journey. Up to 50% of this population survive with new chronic diseases and/or permanent disability, and within a year over half will suffer hospital readmission^4–9^. Physical, psychological and cognitive aspects of recovery, described as ‘post intensive care syndrome’ (PICS), result in greater dependence on health and social care for up to 5 years post discharge^10–12^.

Follow-up care for survivors influences recovery by identifying unmet needs and are included in UK national rehabilitation guidelines and provision of care standards^13,14^. However, findings from large randomised control trials (RCT) and meta-analyses examining the impact of follow up services have demonstrated little effects on outcomes, with one large RCT finding worsening quality of life^15–17^. This highlights the complexities of determining the best service provision for critical care survivors, as they transition across different funding streams and models of care between hospital, community and social care teams. Community teams are often unfamiliar with the critical care recovery process^18^. Secondary care teams need to interact and build collaborations effectively with community and social care teams to understand service availability. This places demand on their time acting as patient advocates^19^. As the number of critical illness survivors grow, so too does the demand to ensure a smooth transition of care from hospital to community and social care teams.

The post-critical illness recovery journey has been well documented from the patient and family/carers perspective internationally, with syntheses highlighting themes such as uncertainty, altered identity and fragmented support^20–26^. Insights on UK services have been limited, focussing on COVID-19 recovery^27–29^. However, these accounts largely represent survivorship as experienced by patients and families alone. To date, there is limited qualitative research that examines how clinicians perceive survivorship and the care delivery for these patients, and how patient and clinician perspectives converge or diverge when navigating post-discharge care systems.

The aim of this study was to qualitatively explore and describe patient and clinician experiences on recovery post-critical illness, identifying common or divergent themes related to navigating care.

## Methods

### Design

A single-centre constructivist qualitative study utilising semi-structured interviews as its methodological approach, nested within a feasibility and acceptability evaluation of a pilot follow-up service for critical care survivors. This study was based at the Royal London Hospital, UK, which has a large adult critical care unit serving a mixed patient population including trauma and neurocritical care patients. The pilot follow-up service augmented the existing clinic by identifying patients’ needs earlier in the recovery process. These needs were identified through the use of patient-reported outcome measures and addressed by a care navigator, who responded by signposting or making referrals to appropriate services. All critical care patients had access to the service, while the clinicians interviewed participated in the existing follow-up clinic. Semi-structured interviews were conducted either virtually or in-person depending on participant preference, with patients and clinicians involved in the recovery pathway. The interviewer was known to the participants, meeting the patient participants as their care navigator and working alongside the clinician participants in the existing critical care follow-up clinic. Topic guides were developed by the study team (supplementary material file 2), with input from the patient and public involvement group, and included exploration of the post-discharge recovery process. This study was conducted in accordance with the ethical principles outlined in the Declaration of Helsinki, and ethical approval was obtained from the Health and Care Research Ethics Committee (reference number 23/YH/0146, 22.08.2023).

### Sampling and recruitment

Patients were purposively sampled to participate in the interviews, based upon the following criteria: age, sex, ethnicity, primary language spoken and reason for admission. Patient participants were approached once discharged from critical care in person on the ward and provided with time to review the patient information sheet (PIS). A member of the study team (SN) approached patients directly to invite them to participate. Although detailed in the PIS, it was reiterated at this time that if the patient declined the invitation to participate it would not affect their ongoing treatment in any way. Those that agreed to take part in the study were invited for telephone or video interview, scheduled for once discharged from hospital. Clinicians were purposively sampled based on their profession and were invited to participate directly by a member of the study team. Written informed consent was obtained for all participants. Sampling continued until the target 20 patients and 10 staff was achieved or until sample judged as adequate using information power^30,31^.

### Data analysis

Interviews were audio recorded and transcribed verbatim (SN). Transcripts were analysed following reflexive thematic analysis principles^30,31^. Analysis commenced after three interviews, with study team members (SN, GM, TS) initially independently coding and then collaboratively developing the code-book to be used for subsequent data analysis (SN, GM), whilst remaining open to identification of new codes. The codebook was used as a flexible and evolving tool to support shared reflexive engagement rather than as a fixed coding framework, consistent with Braun and Clarke’s guidance that teams may draw on shared resources while maintaining an interpretative, reflexive analytic stance. Emergent themes focussing on recovery post critical illness were developed (SN, GM) and then refined by the whole study team in two data meetings^31^. Cross-group comparison of patient and clinician themes was undertaken to identify shared and divergent themes, informing the development of an integrated conceptual synthesis (SN, TS). An audit trail documenting analytic decisions, code development, and theme refinement was maintained throughout. Credibility of the analysis was strengthened through investigator triangulation, with multiple researchers (SN, GM, TS) reviewing and discussing the data and emergent interpretations. Reflexive discussions were embedded throughout the process to consider how researcher positionality shaped analytic decisions. In line with recent critiques of ‘saturation’ within reflexive thematic analysis, we did not use saturation as a definitive endpoint. Instead, sample adequacy was considered through the lens of information power, considering the focused aim of the study, the specificity of the participant groups, the high quality and richness of the interviews, and the systematic, team-based analytic strategy^32,33^. Reflexive team discussions were used to judge when further data collection was unlikely to contribute substantively new insights to the developing analysis, informing the decision to conclude recruitment with 16 survivors and 7 clinicians. No qualitative data analysis software was used, as the reflexive thematic analysis approach prioritised close, iterative engagement with the data; the dataset was of a manageable size for manual coding, and analytic rigour was ensured through ongoing triangulation and reflexive documentation. Reflexive practice was embedded throughout data collection and analysis, with the team critically reflecting on how their professional backgrounds and assumptions shaped engagement with participants, coding decisions, and theme development. The final code-book and core research team positionality is included in the supplementary material (files 1, 3 and 4).

### Positionality statement

TS is a clinical lecturer, qualitative researcher and critical care nurse. Prior to this study he had no significant experience in, or views about, post-intensive care syndrome or the post hospital recovery process for this patent group.SN is a research physiotherapist who has worked in critical care, in the critical care follow up clinic and, in this study, as a care navigator for critical illness survivors. Until this last role she had little experience of, or strong views regarding, post hospital recovery. SN conducted all patient and clinician interviews (the first 3 peer reviewed by TS). GM is a junior doctor working on the adult critical care unit, who was also involved in recruitment for this study. Prior to this, she had little experience of the post hospital recovery process for this patient group. ZP is a critical care consultant and an expert in post-intensive care syndrome. He did not take part in theming.

## Results

### Participant characteristics

A total of 16 critical care survivors (including one carer to assist) were interviewed within the first 3 months of being discharged from hospital, plus 7 clinicians involved in the critical care follow-up clinic at the study site. Participant characteristics are described in Table 1. Interviews took on average 23 minutes. Participants were recruited between October 2023 and December 2024. Twenty-four patients were approached to take part in the study and provided with patient information sheets. Out of those, two declined due to time constraints, five were lost to follow up and one individual passed away.

**Table 1:** Participant characteristics.

Survivors (*n* = 16)	*n*(%) or median (IQR)
*Age at interview (years)*	
<40	5 (31%)
40–60	5 (31%)
>60	6 (38%)
*Sex*	
Female	7 (44%)
Male	9 (56%)
Length of stay in ICU (days)	12 (7-15.5)
*Reason for admission*	
Medical	7 (44%)
Surgical	9 (56%)
*Ethnicity*	
Asian	2 (13%)
Black	4 (25%)
White	10 (62%)
*Primary Language Spoken*	
English	12 (75%)
Bengali	1 (6%)
Other	3 (19%)
Clinicians (*n* = 7)	n(%)
*Profession*	
Nurse	2 (29%)
Physiotherapist	1 (13%)
Psychologist	2 (29%)
Doctor	2 (29%)

### Recovery process themes from interviews

**Fig. 1:**
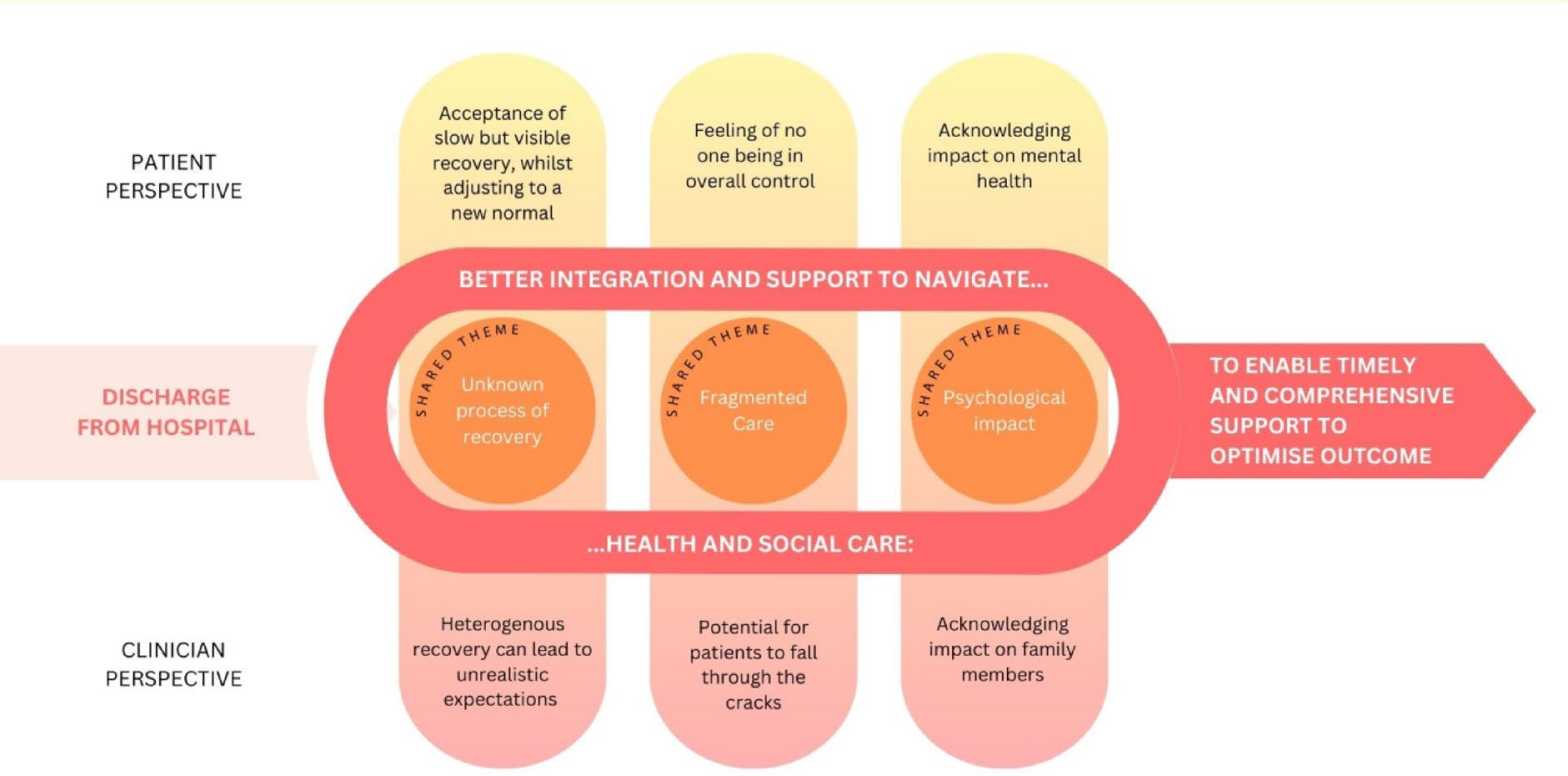
Conceptual synthesis of shared themes.

Key patient and clinician themes are summarised below with supporting quotes (Tables 2, 3). Figure 1 was inductively developed from these themes to produce a conceptual synthesis, reflecting shared perspectives across participants. It is intended as a conceptual illustration grounded in the developed themes rather than a formal theoretical model. The figure emphasises the need for improved integration of health and social care services to support critical care survivors whilst they navigate recovery.

**Table 2:** Summary of key patient themes with supporting quotes.

Patient themes	Quote #	Supporting quotes
Theme 1: Acceptance of slow recovery process as long as some improvement is visible	Quote 1	“You know I just thought it would be you’re out. Everything’s fine, but it’s. It does take time and it’s it’s it’s [sic] a slow recovery…just taking this step by step” (P12)
	Quote 2	“But it’s still a longer process, and I would say, probably everything I do now takes me. It used to take, probably 3 times as long as before. Now probably twice as long, so I guess there is a progress there.” (P1)
	Quote 3	“The curve of recovery, for each injury is slightly different. But luckily, you know, there’s always something getting better. So even if I haven’t seen, for example, my elbow, I could tell that I am stronger generally. I can start walking. I can start exercising every day you notice that something gets a little bit better. Sometimes it’s just overall. You just feel less tired” (P1)
	Quote 4	“I found it difficult…I was getting out of breath…but I kept walking each day to build it up” (P8)
Theme 2: Getting used to a new level of “what is normal for me”	Quote 5	“To some extent my world has shrunk…So you know, you kind of very much limit the range of what you can do. And you know, kind of within that like range, you can function. But it’s a limited range comparing to my activities pre accident” (P1)
	Quote 6	“It’s that difference between a realistic recovery and what’s going to get better and what’s forever going to stay the same. So it’s not realistic for me to be in the same position I was in before because of the spinal cord injury. So some of the times the word recovery was a bit ambiguous in terms of where it was aiming for” (P9)
	Quote 7	“….which I’m finding it extremely hard to do, because I’m back home right now, and I’ve got to be a parent again” (P13)
Theme 3: Access to support (including aids/adaptations) can facilitate independence, function and recovery	Quote 8	“I am thankful for their {physiotherapists} help they put me on the right course and gave me that little bit of confidence which is you know what you really need.” (P5)
	Quote 9	“I’m writing important things down now on the safety notes.” (P13)
	Quote 10	“My recovery, I mean, has been hampered by me not getting to see, I mean, the specialist I needed to see” (P13)
Theme 4: Acknowledging the impact that critical illness has during admission, discharge and recovery on mental health of patient and family	Quote 11	“To be honest, I don’t want to go back there. You know. It makes me so anxious just thinking about it.” (P1)
	Quote 12	“It nearly caused the disruption in the family… I don’t think he realised what he was taking on…I was left alone in a little room downstairs, and they were mostly being upstairs. I just felt, I just felt really, really terrible. I really thought I was going to die in my own son’s house” (P2)
	Quote 13	“She witnessed the whole thing, and I’m more concerned about her… my daughter’s responding quite good to the sessions.” (P13)
	Quote 14	“Although it’s a physical thing, it’s also a mental thing, you know the recovery is also in your head” (P5)
Theme 5: Frustrations with fragmented care (including patient feeling not in control)	Quote 15	“For me, it’s been a really exhausting part of recovery, that, that sense that you’re on your own, and you’ve got to fight for a lot of things……Because there doesn’t seem to me to be anyone in overall charge of things. It’s quite fragmented. And you don’t get the sense that anybody’s in overall control, which is me, me, I’m the one in overall control, allegedly” (P9)
	Quote 16	“You’re not always in a fit state to keep track of where it’s all gone …The earlier on the worse you was so for a good half of that I wasn’t even fit to realise what was going on” (P7)
	Quote 17	“…they did MRIs and everything, but they couldn’t, they couldn’t do any reports on it, because they didn’t have anything to compare with…I don’t think the two hospitals were able to talk to each other” (P2)
	Quote 18	“The doctor did not even ask me why did I come to the surgery? ….not even asking me any question. Or ask me if I have any question for him” (P6)
	Quote 19	“There’s quite there’s, there’s a lot of opportunities for people to fall through the cracks” (P9)

### Theme 1: Acceptance of slow recovery process as long as some improvement is visible

Most participants discussed how their recovery had been a slow process (Quote 1) with one participant stating how this felt particularly true when compared to recovery after non-critical care hospital admissions. Participants described the need to change expectations of how to measure recovery (Quote 2) and how a noticeable improvement in a functional task was extremely encouraging however small (Quote 3). Fragmentation of services isolates patients from healthcare professionals with experience in managing this. Participants talked about the impact of ongoing symptoms, such as pain and fatigue, and concerns regarding how scars have affected appearance. For the majority, these ongoing symptoms were accepted as part of the process (Quote 4).

### Theme 2: Getting used to a new level of “what is normal for me”

A recurrent theme was acknowledgment of a “new normal”. When judging their level of recovery, participants were required to decide whether they were measuring progress or comparing how they were before they were admitted to hospital. This differing timepoint for some solicited positive results as they could see that they will get back to their previous level of “normal”. For others, this led to a feeling of acceptance of new limitations (Quote 5) or a change to previous activities they enjoyed, now perceived as too risky due to the possibility of further injuries. Others discussed how there was ambiguity in the word recovery, with some aspects a temporary “normal” and others now their new “normal” (Quote 6).

This adaptation to a “new normal” was anxiety producing for some, leading to reduced social interactions. Some participants attributed this anxiety to the uncertainty of how much recovery will happen. Others discussed the expectation from those around them to return to their previous level of “normal”, whether that be in work or their role in the family (Quote 7). As with the previous theme, fragmentation of service and interactions prevent healthcare professionals from reassuring and helping participants adapt, leading to a missed opportunity in decreasing anxiety.

### Theme 3: Access to support (including aids/adaptations) can facilitate independence, function and recovery

Many felt grateful for the support from community teams, describing how it led to improved confidence and facilitated their independence (Quote 8). Some felt that this care was tailored to meet their specific needs. Others talked about support and encouragement coming from friends and family.

Some participants talked about adaptations they had come up with to manage symptoms to enable participation in personal care or other daily tasks (Quote 9).

There was an acknowledgement amongst the participants that by not accessing the right support or at the right time, their recovery had been negatively impacted (Quote 10) which is affected by the fragmentation of the system.

### Theme 4: Acknowledging the impact that critical illness has during admission, discharge and recovery on mental health of the patient and family

The impact of being critically ill on mental health was a common theme. For some, this was acknowledging the mental trauma of the event itself and the admission to hospital (Quote 11). For others this was linked with the discharge process, where they felt they were discharged too early, feeling unsafe initially, and placing pressure on family members acting as informal carers, some who had underestimated this role (Quote 12). The impact on the family member’s mental health and the support they need during this recovery period was also discussed (Quote 13).

There was an acknowledgment that recovering from a critical illness did not just include recovering from physical injuries but also from psychological ones (Quote 14). The separation of services for physical and psychological support is a further example of fragmentation and its detrimental effects.

### Theme 5: Frustrations with fragmented care (including patient feeling not in control)

While fragmentation is a major theme, it is also a cross-cutting theme. Most patients discussed frustrations with care (or lack of) that they received after they were discharged from hospital. One participant shared how mentally fatiguing this was to become the person with overall control of a complex set of new care needs (Quote 15). This was particularly difficult in the context of managing multiple follow-up appointments, made harder by the fact that this responsibility was running concurrently with recovery from being critically unwell (Quote 16). Others discussed the frustration of dealing with fragmented care providers who seemingly didn’t communicate (Quote 17), with some feeling that there was no communication of information from their critical care stay, leading to disappointing interactions with their GP who from their perspective hadn’t demonstrated an awareness of the recovery process (Quote 18). Worries were expressed that this fragmented care could lead to not getting the support services needed and therefore impacting on recovery (Quote 19).

**Table 3:** Summary of key clinician themes with supporting quotes.

Clinician themes	Quote #	Supporting quotes
Theme 1: Heterogenous nature of recovery from critical illness and changing focus throughout the process leading to risks of potential for mismanagement	Quote 20	“Definitely critical care. You’re very much the acute side of things, basically keeping somebody alive when you’re doing the follow up clinic, it’s much more rehab, psychological support, referrals that have been for, that have not been done, or that have gone missing, things that you wouldn’t really deal with so much in the intensive care setting, I think.” (C1)
	Quote 21	“I imagine that their first thoughts it feels like their first thoughts are the physical but actually the sort of cognitive and psychological seem to make up as much, if not more of the stuff that we talk about [in follow-up clinic]” (C3)
	Quote 22	“…we pick up the pieces 12 months later and realise it’s a bit of a disaster, and they’ve kind of dropped off from all other services” (C2)
	Quote 23	“People kind of fall between the cracks haven’t been picked up by services. And you know they’re struggling much more to get on with things… They’re not able to really seek help unless they go into one of our services, and someone picks them up” (C5)
Theme 2: Unfamiliar nature of surviving from a critical illness can lead to patients and families having limited understanding and unrealistic expectations of recovery process	Quote 24	“…they don’t really understand where they might end up and what they might need to do to get a bit better” (C2)
	Quote 25	“They’re definitely not as aware as then their relatives are more aware of kind of what priority was at that point, and the relative the patient doesn’t necessarily realise how sick they were because they couldn’t see it” (C1)
	Quote 26	“Sometimes patients and their families think that I’ve left intensive care, I’ve left the hospital, I’ve had my rehab, and now I’m going home and it’s going to be fine. But this is where most of the times the problem starts. And when I’m talking about problems and issues I mean fatigue and nightmares and anxiety and you know I think it’s very difficult because it’s the first time that they’re kind of being alone without any healthcare professionals around them. It’s just them and their family and friends. And I think that they don’t realise up to that point that they do still have a long road ahead of them and lots of challenges to face” (C4)
	Quote 27	“Maybe I’ll be able to manage this. I just want to get on with my life. They start going back to work. They start, you know, doing all the usual kind of life events and hobbies, etc. And then they start noticing that there are things that they aren’t they aren’t able to do.” (C5)
Theme 3: Impact on, and lack of support for families	Quote 28	“Something I have been thinking about more recently is about support for families. And that’s kind of related to the fact that often our patients might not have huge amounts of memory of their ICU admission. But their families do, because they were there. They were awake throughout the whole thing. They were the ones who were told that their loved one might not survive or they was, you know, seriously ill that they were, gonna have amputation, or whatever it was.” (C6)
	Quote 29	“I’m fine, you know. I was the patient. I don’t really remember or you know. Actually, I’m getting on okay. But it’s my, it’s my partner or my son or my nephew that found me unconscious. And they’re really struggling” (C5)
	Quote 30	“We kind of ask families how they’re doing, and I think sometimes we do, but then we get a bit stuck, because if they then say actually, I’m not doing so well…. Then we’re a bit like Hmm. Okay, what can we do with you now? …. Because I think nationally, that’s something that’s lacking, anyway, is support.” (C6)

### Clinician themes

Most of the clinicians that were interviewed had a unique standpoint in that they interacted with this patient population at two timepoints in their recovery journey: at the beginning on the critical care unit, and then in the critical care follow up clinic. The time at which patients are seen in this clinic varies from as early as 3 months post discharge from critical care to over a year post discharge.

### Theme 1: Heterogenous nature of recovery from critical illness and changing focus throughout the process leading to risks of potential for mismanagement

Clinicians expressed that the heterogenous recovery process meant that it was difficult to predict the trajectory and have realistic conversations about recovery. They described how there was a difference in focus of recovery on the intensive care unit compared to in follow up clinic. This was described as an initial focus on the physical aspects of recovery to then the more psychological aspects once they have been discharged into the community (Quote 20). They discussed this shift not only from the point of view of managing patients but also for the patient themselves (Quote 21).

This heterogenous nature of recovery places patients at risk of not being identified by the right services to address their needs. The need to prevent mismanagement and patients “falling through the cracks” i.e. Fragmentation, was discussed by clinicians as their role in the follow up clinic (Quotes 22, 23).

### Theme 2: Unfamiliar nature of surviving from a critical illness can lead to patients and families having limited understanding and unrealistic expectations of recovery process

Surviving a critical illness is an unfamiliar process to patients. Clinicians described how patients often do not have a clear understanding of recovery trajectory and potential for recovery of function nor how long it will take when discharged from critical care and hospital (Quote 24). Clinicians shared that for many patients this may be because they have little memory of their stay on critical care, and that discussions about expected recovery were primarily had with their family (Quote 25). They also reflected on whether these conversations were even being had on critical care whilst trajectory was still unclear, and then it is unknown what happens once they have been discharged to the ward. Patients sometimes had unrealistic expectations about recovery, with a sense that once they are home they will feel better (Quote 26). Clinicians described how patients may even start to reintegrate back into their previous societal roles, including work, but then acknowledge that they still need support to continue to recover (Quote 27) which is specifically affected by fragmentation.

### Theme 3: Impact on, and lack of support for families

The final clinician theme that emerged was recognition of the impact having a relative in critical care has on the family (Quote 28). The patient themselves may alert their clinician to this (Quote 29) or it is identified in follow up clinic. Clinicians commented on the fact that although they may be identifying support needs for families in practice, nationally there is a lack of support available for relatives of critically unwell individuals (Quote 30).

### Shared and divergent themes

Analysis of the interview data identified three overarching themes that were shared across patient and clinician accounts: unknown process of recovery, fragmented care, and psychological impact (Fig. 1). While these themes were present in both datasets, important differences emerged in how patients and clinicians experienced, interpreted, and responded to these challenges.

Both patients and clinicians described recovery following critical illness as uncertain and heterogeneous. Patients framed this uncertainty in relation to their day-to-day recovery, expressing a need to recalibrate expectations and adjust to a “new normal”. For clinicians, uncertainty was articulated in relation to prognosis and trajectory, with many describing difficulty in predicting recovery or having definitive conversations about future outcomes. This difference in perspective created a divergence in expectation management: patients experienced uncertainty as a lack of guidance, whereas clinicians described uncertainty as an inherent feature of post-critical illness recovery.

Fragmented care was a second shared theme, but again experienced differently. Patients described fragmentation as a sense of being left to coordinate care independently across multiple services at a time of reduced physical and cognitive capacity. Clinicians recognised these same system gaps, expressing concern that patients could “fall through the cracks”. While both groups noted fragmentation, the implications differed: for patients, it translated into a sense of being left to manage on their own, whereas for clinicians, it reflected the constraints and limits of responsibility imposed by a complex system.

Psychological impact represented a further shared theme, encompassing emotional distress, anxiety, and the effects of critical illness on family members. Patients described psychological consequences as immediate and ongoing throughout recovery. Clinicians also acknowledged significant psychological burden but described these needs as becoming more visible later in the recovery trajectory, often identified during follow-up clinic appointments rather than earlier transitions of care.

Together, these shared and divergent perspectives informed the development of Fig. 1, which synthesises how uncertainty, fragmentation, and psychological burden intersect across recovery, while highlighting differences in how patients and clinicians perceive responsibility, timing, and agency within the care system.

## Discussion

We set out to explore and describe patient and clinician experiences and views on recovery following a critical illness, identifying common or divergent themes related to navigating the recovery process. By integrating clinician perspectives alongside survivor accounts, this study extends existing literature by moving beyond descriptions of survivorship to examine how shared challenges are interpreted and managed between patients and the clinicians that care for them. While patients and clinicians identified common challenges relating to uncertainty, fragmented care, and psychological impact, key divergences were evident in how these challenges were perceived, when they were recognized by clinicians and how responsibility for care was attributed. We found patients experienced uncertainty, fragmented care, and psychological distress as immediate, ongoing challenges marked by a lack of guidance and support, whereas clinicians described these same issues as reflecting the inherent unpredictability of recovery, delayed presentation of psychological need, and constraints imposed by complex care systems. These divergences draw attention to differences in how recovery challenges are perceived across patients and clinician, highlighting the importance of examining recovery as shaped by interactions between stakeholder groups and care pathways to more effectively inform service improvement.

Here is a wealth of published data describing patients experiences of recovery following critical illness^20–29^. Many of the themes identified in this study have been reported previously, including the centrality of mental health to recovery, the important role of family and friends and challenges arising from poor co-ordination of care^20,24,26^. Concepts relating to adjustment and vulnerability during recovery, such as adapting to a “new normal” has been described, albeit using different terminology and levels of conceptualisation^21,22,26,27^ Fragmentation of care has similarly been identified as a recurring issue in survivorship research, though without the interwoven ramifications^23,25,26,29^. However, much of this literature has examined patient experiences in isolation, without parallel consideration of how clinicians understand and respond to the same challenges. As a result, uncertainty, fragmentation and psychological burden are often described as discrete issues rather than as interwoven features of a recovery system that requires active navigation. By integrating patient and clinician perspectives within a single analytic framework, our findings highlight how shared challenges are interpreted differently across stakeholder groups, and how misalignments in expectations, timing, and perceived responsibility may contribute to persistent difficulties in operationalising recovery support after critical illness.

### Implications for clinical practice

The heterogeneous nature of the patient population, their social circumstances, and their medical conditions result in a variation in response to surviving a critical illness. This in turn leads to heterogenous symptoms that survivors face, exacerbated by fragmented care systems across secondary, primary and community care. The result is that survivors struggle to receive the support they need, when they need it. Better integration and support to navigate health and social care is needed to ensure timely and comprehensive support. Fundamentally this requires a mechanism to negotiate care across organisational borderlands^34^. The divergent perspectives identified in this study suggest that such mechanisms must explicitly address expectation management, ownership of care coordination, and early identification of psychological need. Operationally this would require (a) bi-directional communication between hospitals and social care services prior to discharge planning, (b) personalised rehabilitation and restitution plans mapped to local community resources and (c) a feedback and audit loop to ensure quality, governance and oversight. Our data offer three clear insights into changes into clinical practise that can help such negotiation. Firstly, that critical care follow-up clinicians play an important role not only as care deliverers, but also as care navigators, a key step in understanding how to improve the effectiveness of the recovery process for this patient group. Secondly, discussions about the future of recovery need to be managed earlier in the process, providing an opportunity for expectation management with patients and family. Lastly, in keeping with the extensive published literature, this population is at risk of developing new physical, psychological and cognitive impairments requiring early identification via a standardised tool and a mechanism to operationalise this at a systems level^7,9,10,35^.

### Implications for future research

Future research should look to address the above three outlined implications for clinical practice by investigating the role of clinicians as care navigators, determining an appropriate tool to identify needs and then understanding how best to operationalise this. One factor highlighted in our data that needs to be considered is the response shift that occurs during recovery, described by the patient participants as an acceptance of the “new normal”. A response shift is a phenomena where a change or an adjustment in the way a patient perceives their quality of life and health status is observed^36^. It can be reflected in patient related outcome measures (PROMS) and thus needs to be taken into consideration when analysing data from PROMS over time, especially if using the results to influence decision making and policy^37^. Although this response shift has been observed in the critical care survivorship literature, we are yet to understand how it may be influenced so that clinicians can support patients to improve their perceived quality of life^21,38^. This in turn may improve discussions about recovery and expectation management, improving communication between clinicians and patients/family. Future research needs to explore the underlying mechanisms of this response shift observed during recovery, so that those supporting critical care survivors (e.g., clinicians, family members, policy makers, researchers) can understand what can be done to influence perceptions of health. Future qualitative research should continue to integrate multiple stakeholder perspectives, rather than examining experiences in isolation, to better understand how misalignments between patients, clinicians, and systems shape recovery trajectories. Future research should include clinicians across different stages of the recovery trajectory, including primary care and community-based services, to better understand shared and divergent perspectives throughout the post-critical illness pathway.

One criticism of the current literature is that research is hospital-centric, suggesting that service providers should look beyond this at the complex matrix of support services and the way in which healthcare and social care interacts^39^. As clinical systems and reimbursement mechanisms differ internationally, there are limits to how much we can extrapolate learnings^40,41^. Research into this field therefore needs to be country and situation specific, with future research focussing on the interaction between health and social care and how that impacts on the recovery experience. This will help clinicians and critical care survivors navigate these systems enabling timely access to care. Without this the responsibility will continue to sit with patients and family members to navigate across disjointed systems, leading to unrealistic expectations and frustrations with fragmented care^42^.

### Strengths and limitations

To our knowledge this is the first study combining the clinician and patient perspectives and examining key commonalities and differences. These data build on a body of work exploring perspectives to assist development of systems which enable clinicians to support patients in navigating health and social care teams. The main limitations of this study are that it is single centre, leading to a small sample size of clinicians and that it lacks the voice of the family/carer, an important insight to comprehensively understand this recovery process^43^. Multi-centre research is needed to enhance our understanding of how this process can be improved at a multi-system level, ensuring funding to include the carer’s voice alongside patients and clinicians. Clinician interviews were limited to those working in the follow-up clinic, which may not fully capture perspectives of healthcare professionals involved at other stages of the recovery trajectory, such as in primary care or community services. Future work should include clinicians across these settings to gain a more comprehensive understanding of shared and divergent experiences. The interviews with survivors were modest in duration, which may have reduced the opportunity for some participants to explore all aspects of their recovery in depth. However, participants were always given space to speak freely, and the interviewer did not cut short or guide them away from topics they wished to discuss. The decision to keep interviews shorter was intentional, to minimise burden, strain and fatigue among individuals still in the early stages of recovery after critical illness. All patient interviews were conducted within the first three months after hospital discharge, capturing important early recovery experiences but not the longer-term adjustment processes that may evolve over time. We note that participants’ experiences may have been influenced by the additional support provided by the care navigator, and recovery may be even more challenging for patients without such support, though the extent of this effect is unknown. Additionally, the interviewer was known to participants, having met patient participants as their care navigator and worked alongside clinician participants in the existing critical care follow-up clinic. We acknowledge that these dual roles may have influenced data collection and interpretation. To manage this reflexively, the research team engaged in ongoing discussions about potential biases, critically reflected on how prior relationships and professional perspectives could shape questioning and analysis, and maintained an audit trail documenting analytic decisions. Future research could consider using independent interviewers to further minimise potential influence from prior relationships.

## Conclusion

Our data highlight the variability in recovery experiences, the need for early discussions about recovery expectations, and the frustrations and potential risks with a fragmented care system. By examining both patient and clinician perspectives in parallel, we were able to identify divergences in expectations, perceived responsibility, and timing of support that may not be evident when exploring either perspective alone. These findings indicate a pressing need for systemic improvements in follow-up care and the integration of social and healthcare services to ensure that patients receive timely and comprehensive support.

## Supplementary Information

Below is the link to the electronic supplementary material.

Supplementary Material 1

## Data Availability

The data that supports the findings of this study are available, in an anonymised manner, from the corresponding author upon reasonable request.
